# An Overview of Systematic Reviews of the Role of Vitamin D on Inflammation in Patients with Diabetes and the Potentiality of Its Application on Diabetic Patients with COVID-19

**DOI:** 10.3390/ijms23052873

**Published:** 2022-03-06

**Authors:** Christiano Argano, Raffaella Mallaci Bocchio, Marika Lo Monaco, Salvatore Scibetta, Giuseppe Natoli, Attilio Cavezzi, Emidio Troiani, Salvatore Corrao

**Affiliations:** 1 Department of Internal Medicine, National Relevance and High Specialization Hospital Trust ARNAS Civico, Di Cristina, Benfratelli, 90127 Palermo, Italy; chargano@yahoo.it (C.A.); raffaellamallacibocchio@gmail.com (R.M.B.); marika.lomonaco@hotmail.it (M.L.M.); salvusxyz@gmail.com (S.S.); peppenatoli@gmail.com (G.N.); 2 Eurocenter Venalinfa, 63074 San Benedetto del Tronto, Italy; info@cavezzi.it; 3 Cardiology Unit, State Hospital, Social Security Institute, 20, 47893 Cailungo, San Marino; emidio.troiani@iss.sm; 4 Department of Health Promotion Sciences, Maternal and Infant Care, Internal Medicine and Medical Specialties [PROMISE], University of Palermo, 90127 Palermo, Italy

**Keywords:** vitamin D, diabetes, COVID-19, overview, systematic review, immune system, inflammation, cytokines

## Abstract

Almost two years have passed since the outbreak reported for the first time in Wuhan of coronavirus disease 2019 (COVID-19), due to severe acute respiratory syndrome (SARS)-CoV-2 coronavirus, rapidly evolved into a pandemic. This infectious disease has stressed global health care systems. The mortality rate is higher, particularly in elderly population and in patients with comorbidities such as hypertension, diabetes mellitus, cardiovascular disease, chronic lung disease, chronic renal disease, and malignancy. Among them, subjects with diabetes have a high risk of developing severe form of COVID-19 and show increased mortality. How diabetes contributes to COVID-19 severity remains unclear. It has been hypothesized that it may be correlated with the effects of hyperglycemia on systemic inflammatory responses and immune system dysfunction. Vitamin D (VD) is a modulator of immune-response. Data from literature showed that vitamin D deficiency in COVID-19 patients increases COVID-19 severity, likely because of its negative impact on immune and inflammatory responses. Therefore, the use of vitamin D might play a role in some aspects of the infection, particularly the inflammatory state and the immune system function of patients. Moreover, a piece of evidence highlighted a link among vitamin D deficiency, obesity and diabetes, all factors associated with COVID-19 severity. Given this background, we performed an overview of the systematic reviews to assess the association between vitamin D supplementation and inflammatory markers in patients with diabetes; furthermore, vitamin D’s possible role in COVID-19 patients was assessed as well. Three databases, namely MEDLINE, PubMed Central and the Cochrane Library of Systematic Reviews, were reviewed to retrieve the pertinent data. The aim of this review is to provide insight into the recent advances about the molecular basis of the relationship between vitamin D, immune response, inflammation, diabetes and COVID-19.

## 1. Introduction

In late December 2019, the first pneumonia cases caused by severe acute respiratory syndrome coronavirus 2 (SARS-CoV-2) occurred [1]. By 11 March 2020, the WHO (World Health Organization) had declared the COVID-19 outbreak a global pandemic [2]. Up to 1 February 2022, 378,716,963 cases and 5,675,536 deaths were reported across the world [3]. This disease includes asymptomatic forms and different clinical manifestations [4]. Because of the rapid spread and high mortality rate of COVID-19, major efforts have been made to evaluate the possible risk factors affecting the progression of the disease in COVID-19 patients [5]. Elderly subjects and patients with comorbidities, such as diabetes mellitus (DM), hypertension, cardiovascular disease, chronic obstructive pulmonary disease and chronic renal insufficiency, are more likely to suffer from severe COVID-19, showing a higher mortality rate [6,7,8,9,10]. Overall, DM represents one of the most relevant chronic diseases that impacts hospitalization and mortality rates, with relevant economic repercussions on health care systems. It has been estimated that, in 2019, the prevalence of DM among adults was 463 million worldwide [11]. This disease represents the second most-frequent comorbidity in subjects affected by severe COVID-19 infection, after hypertension [12]. Different studies highlighted that DM is associated with disease severity, poor prognosis and mortality among COVID-19 patients [13,14,15]. However, systemic inflammatory response, increased coagulation activity, immune response impairment and direct pancreatic damage by SARS-CoV-2 might underpin the association between diabetes and COVID-19 [13,16,17]. It was demonstrated that elevated levels of pro-inflammatory cytokines were present in patients with severe COVID-19 and DM [18]. Moreover, a higher expression of ACE2 in human lung tissue has been associated with DM and its related treatments, which might increase sensitivity to SARS-CoV-2 infections [19]. Lastly, the level of glycated dysfunctional hemoglobin may contribute to the hypoxia in COVID-19 patients, most often those who are anemic, at later stages [20]. Therefore, it could be hypothesized that the combination of SARS-CoV-2 infection and DM might represent a negative condition that tends to complicate the course of the disease [21].

Vitamin D is a well-known hormone with principal effect on bone health, but different data has shown the extra-skeletal activities of vitamin D, such as the anti-inflammatory, antioxidant and immunomodulatory ones [22]. Many observational and epidemiological studies have suggested an association between vitamin D insufficiency and the incidence of type 1 and type 2 DM [23,24,25,26]. Furthermore, an intake of high monthly doses of vitamin D proved efficacious in reducing c-reactive protein (CRP) levels.

In fact, COVID-19 is characterized by high levels of inflammatory markers, including CRP, and increased levels of inflammatory cytokines and chemokines [27,28]. Given this background, we conducted an overview of the available systematic reviews to summarize the current knowledge and evidence about the role of vitamin D on the inflammatory process in patients affected by DM. Moreover, based on literature data, we aimed to indicate the possible role of vitamin D supplementation in the setting of patients affected by COVID-19.

## 2. Materials and Methods

### 2.1. Eligibility Criteria

All the meta-analyses and systematic reviews (SRs) regarding the association between vitamin D supplementation and inflammatory markers in patients with diabetes were eligible for this review.

### 2.2. Search Methods

On 13 September 2021, at 22:00 p.m. (GMT-5, Bethesta, MD, USA), a literature search was performed within the database of MEDLINE, PubMed Central and the Cochrane Library of Systematic Reviews (CLSR); the following search strings were used: (“vitamin D” [MeSH Terms] OR “vitamin D” [All Fields] OR “ergocalciferols” [MeSH Terms] OR “ergocalciferols” [All Fields] OR (“ergocalciferols “[MeSH Terms] OR” ergocalciferols “[All Fields] OR” ergocalciferol “[All Fields]) OR (“cholecalciferol “[MeSH Terms] OR” cholecalciferol “[All Fields] OR” cholecalciferols “[All Fields] OR” colecalciferol “[All Fields]) OR (“calcitriol “[MeSH Terms] OR” calcitriol “[All Fields] OR” calcitriols “[All Fields])) AND (“inflammation “[MeSH Terms] OR” inflammation “[All Fields] OR “inflammations” [All Fields] OR “inflammations” [All Fields] OR (“inflammatories” [All Fields] OR “inflammatory” [All Fields]) OR “TNF” [All Fields] OR (“interleukine” [All Fields] OR “interleukines” [All Fields] OR “interleukins” [MeSH Terms] OR “interleukins” [All Fi elds] OR “interleukin” [All Fields]) OR (“cytokin” [All Fields] OR “cytokine s” [All Fields] OR “cytokines” [MeSH Terms] OR “cytokines” [All Fields] OR “cytokine” [ All Fields] OR “cytokinic” [All Fields] OR “cytokins” [All Fields]).

### 2.3. Study Selection

Two authors (RMB and MLM) independently reviewed the titles, abstracts and full texts of the retrieved articles, to determine their potential inclusion using the eligibility criteria. Any disagreement was resolved by discussion with a third author (SC), and, when only limited information was available, the authors of the study were contacted to request the full text or further details.

### 2.4. Data Extraction, Coding and Analysis

**Figure 1:**
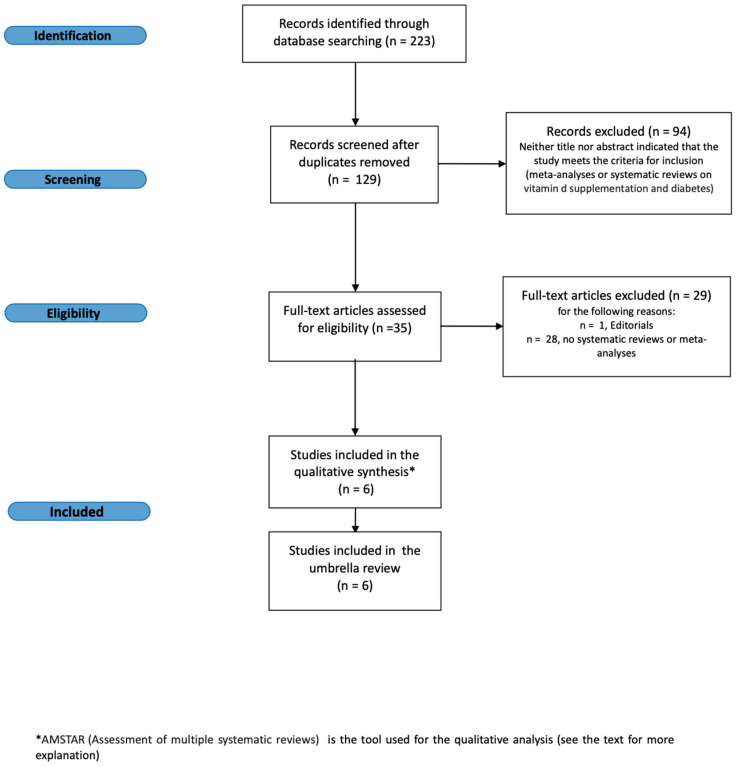
Flow diagram of the study selection process.

Two authors (RMB and MLM) collected data from all the included articles using a pre-tested form and individuated duplicates and prepared the flow-chart of the excluded and included studies. SC and CA independently verified the entire process (Figure 1).

### 2.5. Quality Assessment of the Included Systematic Reviews

Two authors (RMB, MLM) assessed the quality of the included SRs by means of AMSTAR (a measurement tool to checklist assess systematic reviews), which is a validated tool to assess the methodological quality of SRs. It includes 11 domains, such as the a priori protocol documentation, scientific quality and risk-assessment publication bias. Based on AMSTAR evaluations, we ordered the derived scores into tertiles and classified the methodological quality of each review in three categories: “high” (8–11 points out of 11), “moderate” (4–7 points) and “low” (3 or fewer points). (Table 1).

## 3. Results

The data of patients with DM were extracted and tabulated according to the result of each trial. Figure 1 shows the flow diagram of the study selection process. To ensure the highest data recruitment possible, initially we searched all the papers regarding vitamin D and cytokines; thereafter we extracted data about the patients with diabetes. The search strings we used permitted us to retrieve 223 bibliographic citations. These were screened, and 129 papers fit the eligibility criteria. Then, ninety-four studies were excluded because they were duplicates, six papers were removed because they were not systematic reviews or meta-analysis and twenty-nine articles were not taken into consideration as they were not pertinent to the aim of our overview. Thus, we finally identified six relevant papers, which were investigated for the details that pertain to this overview of the SRs. Table 1 shows the summary of the AMSTAR assessment. No SR regarding the vitamin D had low-quality results. Hence, we extracted each randomized clinical trials including patients with diabetes from every systematic review. The data from each randomized clinical data are reported in Table 2. 

The SRs that have been taken into consideration were heterogenous in different aspects: patient characteristics, type of treatment, end-points and measured variables. However, this heterogeneity enriched the final analysis. Six SRs were included, with a follow-up duration between six and fifty-two weeks. Both patients with diabetes type 1 and type 2 were represented. The vitamin D doses used in these studies were widely variable. Three out of six SRs demonstrated a clear efficacy of the supplementation as to CRP reduction; one SR showed a statistically significant reduction of interleukin-6 (IL6) with vitamin D intake. Table 2 shows the various dosages used in each study. In their systematic review, Asbaghi et al., analyzing the only randomized clinical trial including subjects with type 2 diabetes, showed that 50,000 IU/week of vitamin D determined a reduction of serum CRP concentrations (−1.19 μg/mL ± 0.25); similarly, Chen and colleagues found that 1000 IU/day decreased CPR levels [CPR −0.40 (−1.12, 0.31]. According to Yanting Yu et al., the vitamin D supplementation significantly decreased the hs-CRP level by an average of 0.45 μg/mL [*p* = 0.005], particularly using a daily dose ≤4000 IU with a supplementation time >12 weeks [*p* = 0.008]. On the contrary, these authors showed no impact of the supplementation on TNF-α and IL-6 concentrations. On the other hand, Mazidi et al. showed that vitamin D supplementation had no impact on CRP, IL-10 and TNF-α, whereas IL6 serum levels were detrimentally and significantly increased by the administration of vitamin D [0.67 pg/mL)]. Agbalalah and colleagues reported that, in patients with type 2 DM, a single-dose administration of 100,000 IU of vitamin D2 resulted, after 8 weeks, in a significant improvement of endothelial function, measured by flow-mediated dilation (FMD) [increase of 2.35 ± 3.12% in FMD from a baseline of 6.38 ± 4.31% (*p* = 0.048)]. According to Fisher et al., in patients with type 1 diabetes, different doses of vitamin D caused a higher proportion of T regulatory cells in comparison with controls [+6.4% (DS 0.8%) of CD4+CD25+CD127, +4.55% ± 1.5% of CD4+CD25+Foxp3+, +22.2 ± 47.2% of CD4+CD25hi FoxP3+CD127low in CD4+ cells, respectively].

## 4. Discussion

SARS-CoV-2 is the novel coronavirus agent of the severe acute respiratory syndrome that brought about the COVID-19 pandemic [35]. The CoV genome encodes four main proteins: spike, membrane, nucleocapsid and envelope [36,37]. The virus’ spike protein is responsible for the virus entry into host cells by recognizing and binding to a few receptors, such as ACE2, CD147 and sialic acid molecules. The viral spike protein that binds to the cell membrane receptors is proteolyzed by the transmembrane serine protease 2, which facilitates its entry into target cells [38,39]. Once in the cell, the viral RNA genome is released into the cytoplasm to begin its replication process [40]. The virus can negatively regulate the expression of ACE2, leading to the upregulation of angiotensin II (Ang II). Ang II interacts with Ang II type 1 receptor (AT1R) to regulate nuclear factor-κb (NF-κB) signaling pathways, as well as the activation of macrophages, which leads to an overproduction of pro-inflammatory cytokines [41]. The key factor, in this positive feedback loop is IL-6, which causes cytokines to be released out of control [42]. IL-6 is an important functional marker of cellular senescence, and the age-dependent increase in IL-6 amplifier may correspond to the age-dependent increase in COVID-19 mortality [42,43]. More recent studies have also unveiled potential roles of ACE2 in regulating immune responses rather than simply being a viral linkage receptor in COVID-19 [42,44,45,46,47,48]. Once ligated to SARS-CoV-2, the expression of ACE2 on the host cell surface was significantly decreased. IL-6 in toll-like receptor signaling pathway could influence the immune system as a downstream effector [42,44,45,46,47,48]. Dysregulation of ACE2 induced by SARS-CoV-2 infection may further cause cytokine storms and pneumonia. Many more detailed pro-inflammatory and detrimental phenomena have been equally described, based on the interaction of SARS-CoV-2 and CD147 and sialic acid membrane receptors [20,49,50,51,52,53]. This increased cytokine production is usually defined as “cytokine storm”. It also triggers a pathogenic inflammatory immune response, leading to severe multi-organ failure and death in patients with COVID-19 [54,55,56].

### 4.1. Cytokine Storm

Cytokine storm denomination has been used to describe hyperactive immune responses that can be initiated by a variety of factors, such as viral infections, autoimmune diseases and immunotherapies [57,58,59] Cytokine storms lead to the elimination of pathogenic microorganisms but also cause tissue toxicity affecting different organs [54,60]. Cytokine release syndrome (CRS), a type of systemic inflammation syndrome caused by cytokine storm, has been previously reported in patients infected with SARS-CoV and MERS-CoV. During viral infection, damage-associated molecular patterns (DAMPs) and pathogen-associated molecular patterns (PAMPs) can activate antiviral responses in nearby cells and recruit innate and adaptive immune cells, such as macrophages, natural killer (NK) cells and gamma delta (gd T) cells (Figure 1) [61,62,63,64,65,66]. Downstream production of interferons promotes intracellular antiviral defenses in neighboring epithelial cells. These may limit viral spread, whereas the release of IL-6 and IL-1b from other immune cells invokes the recruitment of neutrophils and T cells [62]. T-cell activation or immune cell lysis prompts the secretion of IFN-g and TNF-a, leading to the activation of immune cells and endothelial cells, with the further release of pro-inflammatory cytokines in a positive feedback loop [60]. These inflammatory mediators may promote thrombus formation [66]. This process, called immune-thrombosis, can also amplify cytokine production, and is illustrated by the binding of thrombin to inflammasome activation and IL-1 production [67]. Because vascular endothelial cells are exposed to circulating cytokines and other immune mediators, coagulation defects (such as capillary leak syndrome, thrombus formation and even DIC) may also be caused by endothelial cell dysfunction in cytokine storms, highlighting the crosstalk between hemostasis and cytokines [61,66]. The cytokine storm not only limits further spread of the virus but also induces secondary tissue damage through the secretion of massive amounts of active mediators and inflammatory factors [55,56,57,58,59,60,61,62,63,64,65,66,68,69,70]. The inhibition of this self-amplifying inflammatory cascade may not only control tissue damage but also impair viral clearance.

In COVID-19, Huang et al. had noted that patients in intensive care units (ICUs) had higher levels of plasma inflammatory cytokines IL-2, IL-7, IL-10, G-CSF (granulocyte colony-stimulating factor), IFN-γ and MCP, and TNF-α compared to patients not in ICUs [71]. These cytokines not only suggested the presence of Th1 answers but also the presence of Th2 answers in COVID-19. Furthermore, monocyte activation may imply that the cytokine storm in COVID-19 is closely correlated with disruption of the balance between innate and adaptive immunity. Recently, studies also showed that the level of IL-6 in severe cases was markedly higher than that in mild and moderate cases, but the levels of CD4+ T cells, CD8+ T cells and NK cells were decreased, indicating immunosuppression in severe COVID-19 patients [68]. Meanwhile, T lymphocyte cells were over-activated during cytokine storm in COVID-19 patients, which may be accompanied by severe immune dysfunction [72]. In a recent systematic review based on autopsy findings, in lung specimen and other organs, fibrin thrombi associated with increased CD61 positive platelets and megakaryocytes in pre-capillary and post-capillary vessels without complete luminal obstruction were observed in specimens collected from patients with COVID-19 [73]. Thus, a cytokine storm can directly damage the pulmonary capillary mucosa, promote alveolar oedema and further induce the spread of inflammatory cytokines, resulting in damage to alveolar structure and dysfunction in pulmonary ventilation [73,74]. In the same way, cytokine storm is also associated with the sequence and severity of organ dysfunction in multiple organ dysfunction syndrome (MODS) [69]. Hence, cytokine storm may be considered an important factor influencing the fate of patients with COVID-19 multi-organ disease.

### 4.2. Innate and Adaptive Immune Response and COVID-19

Innate and adaptive immune reactions cooperate with each other to produce immune protection [4]. Innate immune responses occur immediately after infection and are typically involved in virus removal, but it has a diminished antiviral capacity. Adaptive immunity is the key factor in the complete eradication of the virus [4]; this immune pathway needs 4 to 7 days to be activated after the occurrence of the infection. If an effective adaptive antiviral response is not generated in time to suppress the virus, innate immune responses will potentiate but are unable to effectively eradicate the virus, and this also leads to systemic inflammatory responses with the irrepressible release of inflammatory cytokines [75,76,77,78]. Elderly patients and those with chronic diseases need a longer period of time to generate adaptive and innate immune responses because of cell senescence. These patients rely only on the enhancement of antiviral innate immune responses in the early stages of infection, generating a higher risk of cytokine storms; thus a more rapid evolution towards severe disease is expected. It is still unclear whether the immune hyperactivity is due to ongoing viral replication or immune dysregulation [66]. Lastly, the NLR family pyrin domain containing 3 (NLRP3) is the most acknowledged inflammasome pattern which takes place in COVID-19, including most of the immune-inflammatory pathways elucidated above.

Interestingly, diabetic patients show an upregulated NRLP3 pathway [79] which could be one of the possible explanations of their susceptibility to this viral infection. At the same time, it was shown that, among the possible compounds that specifically target the NRLP3 inflammasome, vitamin D proved to downregulate this pathway, inhibiting IL-1β secretion in macrophages in vivo [80].

Taken together, viral escape to avoid antiviral immunity, may compromise viral clearance, resulting in inappropriate immune activation and, consequently, causing cytokine storms [81]. Thereby, this activation of innate immunity may be an important factor in the development of cytokine storms in COVID-19 [82].

### 4.3. Vitamin D

Vitamin D, a secosteroid hormone, regulates calcium and phosphate homeostasis but also cell proliferation and differentiation. It plays a vital role in keeping the mineralized skeleton healthy, and it also plays a crucial function in the response of the immune system [83,84]. Experimental and animal studies have shown, firstly, that vitamin D has important biological activities on the innate and adaptive immune system and, secondly, that the administration of vitamin D changes the onset and progression of various immune-factor diseases [85]. Humans get their vitamin D from sunlight, diet and supplements. The two main forms are: vitamin D2 or ergocalciferol and vitamin D3 or cholecalciferol. After entering the circulation, vitamin D (D expresses both vitamin D2 and D3) is metabolized by vitamin D-25-hydroxylase (CYP2R1) in the liver to 25-hydroxyvitamin D or calcifediol [25 (OH) D]. 25 (OH) D is further metabolized, mainly in the kidneys, by the enzyme 25-hydroxyvitamin D-1α-hydroxylase (CYP27B1) into the active form, 1,25-dihydroxyvitamin or calcitriol (CT) [1,25 (OH)2 D] [84,86]. Then, 1,25 (OH)2 D employs its physiological functions by binding to the vitamin D receptor (VDR) in the cytoplasm of cells, stimulating the heterodimerization of the VDR with the retinoid X receptor (RXR), forming a VDR-RXR-hormone complex [15]; in the nucleus, it leads to the up- or downregulation of a multitude of genes [84]. Kidneys are the primary site of the conversion of 25 (OH) D to systemically bioavailable 1,25 (OH)2 D. CYP27B1 is also expressed by many other tissues, including activated macrophages, parathyroid glands, microglia, breast, colon and keratinocytes, wherein 1,25 (OH)2 D is produced and exercises its autocrine and paracrine function [83,85,87].

During an infection, macrophages and monocytes are recruited to the inflammatory site; the exposure to inflammatory cytokines expresses CYP27B1, which converts 25 (OH) D to 1,25 (OH)2 D [88]. Then, 1,25 (OH)2 D develops the antimicrobial activities of macrophages and monocytes. Furthermore, 1,25 (OH)2 D suppresses the expression of toll-like receptors on monocytes and inhibits the production of some inflammatory cytokines such as IL-2, IL-6 and IL-17 [85,89,90]. Experimental studies have also shown that natural killer (NK) cell differentiation and function can be modulated by 1,25 (OH)2 D administration. At present, the data regarding the influence of 1,25 (OH)2 D on NK cells are still inconsistent [91,92,93].

Through multiple genomic and extragenomic pathways, numerous experimental studies have demonstrated that vitamin D and its metabolites modulate endothelial function and vascular permeability [94]. Other studies have indicated that 1,25 (OH)2 D3 is a transcriptional regulator of endothelial nitric oxide synthase (eNOS). This causes the upregulation of eNOS gene expression and consequently, an increase in endothelial nitric oxide production [93,95,96]. In local and systemic inflammation, vitamin D and its metabolites employ pleiotropic effects on vascular endothelium that are protective against vascular dysfunction and tissue damage [97,98]. Vitamin D may also activate hepcidin-antagonist pathways, regulating the hepcidin-ferroportin axis, which can be of help in COVID-19, for which hyperferritinemia is one of the major negative prognostic factors [20]. Vitamin D3 may also have an anticoagulant effect, in contrast to cholecalciferol insufficiency, which may be pro-thrombotic [99]. Recent studies showed that, in intensive care patients, 100,000 IU/day of cholecalciferol for five days resulted in higher and lower levels of hemoglobin and hepcidin, respectively. Cholecalciferol showed upregulatory epigenetic action on a few antioxidant systems too [100].

It was observed that a drastic shift from the proinflammatory state to a more regulated immune-inflammatory activity is achieved as a result of the local production of 1,25(OH)2D by monocytes/macrophages [101]. This is considered one of the reasons why vitamin D might exert protective effects against autoimmune diseases. Other studies have also demonstrated that a decreased CD4/CD8 ratio was associated with low 25(OH)D levels [102] and that the administration of 5000–10,000 IU of vitamin D3 was attributable to an increase in the CD4/CD8 ratio, reflecting immune regulation [103,104].

As regards B lymphocytes, inactive B lymphocytes do not have VDRs but only upregulate their VDR expression when they are activated to proliferate with mitogens [105]. Furthermore, 1,25(OH)2D inhibits immunoglobulin synthesis and therefore could potentially interfere with the immune system, and 1,25(OH)2D also regulates B-cell activity. The hyperactive state of 1,25(OH)2D appears to attenuate the immunoglobulin immune response through a variety of mechanisms [105,106,107,108,109].

By controlling B cell activity and the transformation of B cells into plasma cells, 1,25(OH)2D contributes to a reduction in autoantibody production, resulting in a reduced risk of antibody-mediated autoimmune disorders [99,104,105,110].

### 4.4. The Link among Diabetes, Vitamin D and COVID-19 Pandemic

It is well-known that people with diabetes are at higher risk of infections [111,112]. Diabetes is characterized by a hyperglycemic environment that promotes immune dysfunction through a variety of ways. In particular, in patients with DM, monocytes and mononuclear cells secrete less interleukin 1 (IL-1) and IL-6 when stimulated by lipopolysaccharide [113,114]. The low production of interleukins seems to be the result of inherent defects [113,115]. Hyperglycemia is also characterized by the reduced mobilization, chemotaxis and phagocytic activity of polymorphonuclear leukocytes [114,116,117]. A hyperglycemic environment blocks antibacterial function by inhibiting glucose 6-phosphate dehydrogenase (G6PD), increases the apoptosis of polymorphonuclear leukocytes and reduces the migration of polymorphonuclear leukocytes through the endothelium [114]. A reduction in C4 is associated with polymorphonuclear dysfunction and reduced cytokine production [111,113]. In addition, a hyperglycemic environment will increase intracellular glucose levels and then the utilization of NADPH as a cofactor for metabolism. The reduction of NADPH levels prevents the regeneration of molecules that play a key role in the cellular antioxidant mechanism, thus increasing sensitivity to oxidative stress. When glycosylated hemoglobin (HbA1c) is more than 8.0%, the proliferation function of CD4 T lymphocytes and their response to antigens by the altered expression of cellular adhesion molecules are affected [114]. Moreover, the virulence of different pathogens can be increased by hyperglycemia [116,117].

Concerning SARS-CoV-2 infection, clinical reports found DM to be one of the most-common comorbidities present in patients exhibiting a more severe course of the disease [118]. Generally, the susceptibility to the viral action seems to depend mainly on the typology/expression of the host cell receptors and on the affinity of the spike with these receptors. Interestingly, diabetic (and obese) patients show an overexpression of CD147 receptors [20], of ACE2 molecules [119] and, especially, the altered glycosylation of all membrane receptors [120,121]. Lastly, hepcidin axis upregulation has been detected in diabetic patients [122], which reinforces the likelihood of intracellular ferritin overconcentration in these individuals.

Overall, this altered profile of cell membrane receptors in DM may be one of the main factors explaining the higher susceptibility of these patients to COVID-19.

A link between hyperglycemia and ACE2r levels and the severity of COVID-19 disease has been documented, probably due to the changes in ACE2r glycosylation and viral spike protein glycosylation. Both may be caused by uncontrolled hyperglycemia, which may modify the binding of the viral spike protein to ACE2r and the degree of immune response to the virus [123].

Elevated blood glucose levels can directly increase the glucose concentration in airway secretions [124]. In uncontrolled hyperglycemia, high and abnormally glycosylated cell receptors in the lungs, nasal airways, tongue and oropharynx may also serve as increased SARS-CoV-2 virus binding sites, resulting in a higher trend of COVID-19 infection and more serious forms of the disease [123]. Glycemic control could reduce the levels of glycosylated ACE2r target in the lung, decreasing the number of glycosylated viral binding sites and possibly ameliorating inflammation and the symptoms of COVID-19 disease [123]. Hyperglycemia may also affect pulmonary function, and this effect could be linked to ACE2r overexpression in the lungs of diabetics [19].

In patients with diabetes, higher circulating glucose levels will result in a higher percentage of glycated hemoglobin. SARS-CoV-2 surface proteins seem to bind to and potentially impair the heme molecule within red blood cells. In this way, a separation of iron from the molecule to form porphyrin occurs, determining in red blood cells an altered oxygen and carbon dioxide carriage, with consequent possible systemic alterations induced by free heme circulation [20,125].

Whereas diabetics and older subjects have more glycated hemoglobin, they may be preferentially affected by SARS-CoV-2 binding and dissociation of iron from heme to form porphyrins, and another receptor (CD147 or basigin) might be involved [49]. Affecting overall hemoglobin functionality, DM may alter oxygen transportation capacity, which may significantly impact the hypoxia patterns of these patients. Different studies reported that diabetic patients have low 25(OH)D levels, which may be due to impaired liver and kidney metabolism of vitamin D, reduced dietary vitamin D intake and decreased intestinal absorption of vitamin D caused by diabetic autonomic neuropathy [126,127,128]. In addition, it has been reported that low circulating 25(OH)D levels are associated with poor blood glucose control in diabetic patients. Prospective studies have shown that vitamin D deficiency may increase the risk of fasting blood glucose impaired and diabetes [129,130,131]. Furthermore, a clear association between hypovitaminosis D, obesity and diabetes mellitus, factors known to increase COVID-19 severity risks, have been widely recognized [126,127,132,133].

It worth to outline that vitamin D deficiency has been hypothesized to predispose individuals to SARS-CoV-2 infection and to increase COVID-19 severity. According to Di Filippo et al. [132], patients suffering from vitamin D deficiency and hyperglycemia were at a higher risk of severe COVID-19, higher inflammatory response and worse disease outcomes.

It was estimated that about 1 billion people worldwide have low vitamin D levels, and this is detected in all ethnicities and age groups [134]. Moreover, a significantly higher prevalence of vitamin D deficiency is reported in DM2 (83.5%) compared to normoglycemic controls in a north Indian community [126]. Tecilazich and colleagues found low 25(OH)D levels in diabetic patients with retinopathy [134], suggesting that hypovitaminosis D may worsen the predisposition of patients with diabetes to the microvascular damage typical of COVID-19. It was shown that supplementation with vitamin D may improve glucose metabolism control by reducing insulin resistance and stimulating β-cell function [135,136], especially in patients with poor baseline blood glucose control [137].

A recent cross-sectional study showed that there is a statistically negative correlation between 25(OH)D levels and the homeostasis model assessment of insulin resistance, but this association was only found in the female population and not in men [138]. Moreover, some studies have suggested that vitamin D treatment may slow the progression to diabetes in patients either at high risk of diabetes or with prediabetes, specifically in those with low baseline 25(OH)D levels [139].

Low 25(OH)D levels may be a predisposing factor in the bidirectional interrelation between diabetes and COVID-19, increasing from one side the susceptibility of diabetics to the infection, from the other side promoting the diabetogenic effect of COVID-19 in terms of endothelial dysfunction and microvascular complications.

Obesity and overweight may equally play a role in COVID-19. High BMI and altered body composition, with increased adiposity, are reported as independent risk factors for greater disease severity and poor prognosis in COVID-19 patients [139,140]. Interestingly, low levels of 25(OH)D were frequently reported in obese and overweight patients, being inversely related to BMI and adiposity [132,141], negatively influencing skeletal and muscle health, with a resulting increased predisposition to an obese osteosarcopenic phenotype [142,143]. In fact, BMI has also been reported to predict resistance to vitamin D [144]. A possible direct relationship between vitamin D status, adiposity, age and COVID-19 severity has been previously hypothesized. In fact, aging and fat accumulation may decrease vitamin D bioavailability and efficacy [29]. A low vitamin D status is present in obese patients and patients with metabolic syndrome, and these conditions are associated with reduced hepatic 25-hydroxylation of vitamin D. Experimental studies showed that CYP2R1 (the major vitamin D-25 hydroxylase) is lower in the livers of obese mice in comparison with normal mice [145]. According to Ekwaru and colleagues obese and overweight subjects had serum 25(OH)D significantly lower than normal weight people and vitamin D supplementation would be 2 to 3 times higher and 1.5 times higher for obese and overweight subjects respectively, in comparison with normal weight subjects [146].

A recent study showed that a strong relationship exists among vitamin D, glycemia and BMI in COVID-19 subjects [147]. Vitamin D deficiency could be identified as a common pathophysiological mechanism involved in the detrimental effect of hyperglycemia and adiposity on disease severity.

Overall, a clear-cut effect of vitamin D serum level on COVID-19 incidence and prognosis was demonstrated [30], which may be explained through the several beneficial effects of this pre-hormone on several biochemical pathways that may putatively contrast the viral invasiveness.

This umbrella review demonstrated that vitamin D supplementation in subjects with diabetes leads to improved circulating inflammatory biomarkers, representing an adjuvant therapy for COVID-19 patients with diabetes and a vitamin D status deficiency. It can therefore be affirmed that, based upon this umbrella review, a strong rationale exists for the therapeutic administration of supplemental vitamin D in order to reduce COVID-19 respiratory complications or prevent, in case of infection, a severe form of COVID-19.

The major strength of this analysis is represented by the resolution of the clinical heterogeneity problem. The presence of clinical heterogeneity across studies is related to the different characteristics and health conditions of the participants included. In this sense, we considered only patients affected by diabetes and according to the different types of diabetes. In addition, our analysis also highlights the heterogeneity of treatments [29,30,31,32,33,34]. In fact, heterogeneity exists in doses of the vitamin administerd to the target population. This study has a limitation. The umbrella review makes a qualitative assessment and compiles all the evidence from existing reviews on a topic through to a specified date. Through extensive searching, an additional thirteen randomized clinical trials [RCTs] are available at this time and, obviously, had not been considered in this umbrella review. For this reason, further studies are necessary for the evaluation of these RCTs. In this sense, it must be outlined that, according to Sabico et al. [148], oral supplementation with vitamin D3 reduces the time to recovery for cough and ageusia among patients with COVID-19, highlighting the beneficial effects of vitamin D supplementation against COVID-19. On the contrary, it is worth it to outline that recent studies showed that long-term supplementation with vitamin D(3) did not reduce IL-6, hsCRP or NT-proBNP in patients with type 2 diabetes [149]; additionally, high-dose vitamin D supplementation did not improve biomarkers of glycemia, inflammation, neurohormonal activation or lipids [150].

## 5. Conclusions

Our overview of systematic reviews concerning vitamin D’s role in the inflammatory processes, highlighted a series of documented interactions among this molecule and a large series of cell metabolic pathways involved in DM and the potential application in patients with diabetes and COVID-19. Current evidence supports the benefits of vitamin D supplementation for managing or treating both of these pathological conditions. Most of the literature reports showed that vitamin D supplementation significantly reduce CRP in diabetic patients, while contrasting data are available about IL-6. Further studies should highlight the optimal treatment doses for the maximum benefit to patients. Meanwhile, vitamin D deficiency should be corrected, since vitamin D supplementation is safe, and it results in potential benefits on the cytokine storm by reducing the severity of several respiratory complications of COVID-19.

## Figures and Tables

**Table 1:** AMSTAR assessment for each systematic review regarding the association between vitamin D supplementation and inflammatory markers in patients with DM. Colors refer to scores: Green refers to “high scores” (8–11 points) and yellow to “moderate” (4–7 points). No systematic review had a “low” (<3 points) evaluation.

		High	Moderate	Low
Vitamin D	Omid Asbaghi (2019) [29]	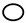	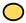	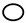
	Sheila A. FisherID (2019) [30]	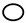	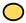	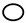
	Yanting Yu (2017) [31]	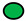	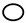	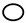
	Mohsen Mazidi (2018) [32]	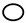	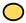	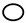
	Tari Agbalalah (2017) [33]	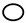	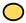	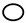
	Neng Chen (2014) [34]	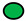	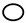	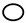

**Table 2:** Clinical trial data extracted for each meta-analysis reporting vitamin D supplementation in patients with diabetes.

Clinical Trial Data Extracted for Each Meta-Analysis Reporting Vitamin D Supplementation in Patients with Diabetes							
Meta-Analysis	Study	Sample Size	Population	Posology	Intervention Duration Range	Endpoint	Efficacy
Omid Asbaghi (2020) [29]	Tabesh 2014	59	Patients with type 2 diabetes mellitus	1000 mg/day Ca carbonate + 50,000 IU/wk Vitamin D3	8 weeks	CRP IL-6 TNF-α	We found a beneficial effect of vitamin D-calcium co-supplementation on serum CRP concentrations while there was no effect on IL-6 and TNF-α
Sheila A. Fisher (2019) [30]	Bogdanou 2017	39	Patients with recent onset of >2 months or chronic type 1 diabetes	4000 IU/day Vitamin D3 for three months (120,000 IU/monthly)	6, 12 weeks	CD4+CD25+ CD127	Vitamin D improves the absolute T regulatory cells numbers and phenotypes in patients with diabetes
	Gabbay 2012	38	Patients with a new diagnosis of type 1 diabetes (T1DM)	2000 IU/day Vitamin D3 for 18 months (60,000 IU monthly)	6, 12, 18 months	CD4+CD25+ Foxp3+ in Peripheral blood	
	Treiber 2015	30	Young patients with new-onset type 1 diabetes	70 IU/kg/day Vitamin D3, weekly for 12 months (loading dose 140 IU/kg/day for 1 month) (4200 IU for one month then 2100 IU monthly)	3, 6, 12 months	CD4+CD25hi FoxP3+ CD127low in CD4+ cells	
Yanting Yu (2018) [31]	Breslavsky 2013	47	Patients with type 2 diabetes mellitus	1000 IU/day Vitamin D3	52 weeks	CRP TNF-α IL-6	Vitamin D supple-mentation is benefi-cial for the reduction of hs-CRP inT2DM subjects but does not have a signifi-cant influence on TNF-α and IL-6 in T2DM subjects.
	Farrokhian 2016	60	Patients with type 2 diabetes mellitus	25,000 IU/week Vitamin D3	26 weeks		
	Asemi 2016	66	Patients with type 2 diabetes mellitus	200 IU/day Vitamin D3	12 weeks		
	Tuomainen 2015	68	Patients with type 2 diabetes mellitus	1600 – 3200 IU/day Vitamin D3	20 weeks		
	Sadiya 2015	82	Patients with type 2 diabetes mellitus	3000 – 6000 IU/day Vitamin D3	12 weeks		
	Gagnon 2014	80	Patients with type 2 diabetes mellitus	2000 IU/day Vitamin D3	26 weeks		
	Tabesh 2014	118	Patients with type 2 diabetes mellitus	50,000 IU/week Vitamin D3	8 weeks		
	Ghavamzadeh 2014	51	Patients with type 2 diabetes mellitus	400 IU/day Vitamin D3	14 weeks		
	Dalan 2016	64	Patients with type 2 diabetes mellitus	2000–4000 IU/day Vitamin D3	16 weeks		
	Jafari 2016	59	Patients with type 2 diabetes mellitus	2000 IU/day Vitamin D3	12 weeks		
	Al-Sofiani 2015	20	Patients with type 2 diabetes mellitus	5000 IU/day Vitamin D3	12 weeks		
	Akbarzadeh 2013	70	Patients with type 2 diabetes mellitus	20 IU/day Vitamin D3	12 weeks		
	Neyestani 2012	90	Patients with type 2 diabetes mellitus	500 IU/day Vitamin D3	12 weeks		
Mohsen Mazidi (2018) [32]	Breslavsky 2013	47	Patients with type 2 diabetes mellitus	1000 IU/day Vitamin D3	6 months	CRP TNF-α IL-6	Vitamin D supplemen-tation had no impact on serum CRP and TNF-α, while significantly increased serum IL6.
	Ohk-Hyun Ryu 2014	50	Patients with type 2 diabetes mellitus	2000 IU/day Vitamin D3	24 weeks		
	Tina K. Thethi, 2015	55	Patients with type 2 diabetes mellitus	1 mcg/day Paricalcitol	3 months		
	Ulla Kampmann 2014	15	Patients with type 2 diabetes mellitus	5600–11,200 IU/day Vitamin D3	12 weeks		
Tari Agbalalah (2017) [33]	Sugden 2008	34	Patients with type 2 diabetes mellitus	100.000 IU Vitamin D3	8 weeks	EF measured by FBF or FMD.	Significant increase of 2.35 ± 3.12% in FMD
	Witham 2010	58	Patients with type 2 diabetes mellitus	100.000–200,000 IU Vitamin D3	16 weeks		No change in FMD at both 100,000 IU and 200,000 IU
	Brevlasky et al. 2013	47	Patients with type 2 diabetes mellitus	1000 IU/day Vitamin D3	52 weeks	CRP	No change in hs-CRP
	Yiu et al. 2013	100	Patients with type 2 diabetes mellitus	5000 IU/day Vitamin D3	12 weeks	FMD and CRP	No change in both endothelial and inflammatory markers measured
Neng Chen (2014) [34]	Breslavsky 2013	47	Patients with type 2 diabetes mellitus	1000 IU/day Vitamin D3	48 weeks	CRP	Vitamin D supplemen-tation is beneficial for the reduction of circu-lating hs-CRP
	Shab-Bidar 2012	100	Patients with type 2 diabetes mellitus	1000 IU/day Vitamin D3	12 weeks	CRP	

CRP = C-Reactive Protein, FoxP3 = Forkhead box P3, TNF-α =Tumor necrosis factor-α IL-6 = interleukin-6, EF = Endothelial Function, FBF = Forearm Blood Flow, FMD = Flow Mediated Dilation.

## Data Availability

Not applicable.
